# Advancements in Postoperative Care after Cataract Surgery

**DOI:** 10.3390/jcm11113162

**Published:** 2022-06-02

**Authors:** Piotr Kanclerz, Raimo Tuuminen

**Affiliations:** 1 Helsinki Retina Research Group, Faculty of Medicine, University of Helsinki, 00014 Helsinki, Finland; 2 Hygeia Clinic, 80-286 Gdańsk, Poland; 3 Department of Ophthalmology, Kymenlaakso Central Hospital, 48210 Kotka, Finland

Cataract surgery is one of the most frequently performed surgical procedures in many countries. The procedure itself is considered safe and efficient with good visual outcomes, and the development of modern surgical techniques and technologies has considerably reduced the frequency of complications. With increased life expectancy, cataract surgery is becoming more common, even among very old patients. As shown in the article by Nussinovitch et al., cataract surgery in very old patients may demand more experienced surgeons due to higher nuclear density and the rates of intraoperative floppy iris syndrome [1]. This is of utmost importance, as one of our earlier studies has shown that, at one of the timepoints, the prevalence of capsular bag complications in a general population undergoing cataract surgery is 7.03% for resident surgeons and only 0.36% for senior surgeons [2]. Elderly patients are also at risk of ocular surface conditions including blepharitis, which is the most frequent cause of cataract surgery cancellation since it might be a primary risk factor for endophthalmitis [3]. The article by Nowomiejska et al. extensively analyzed the prevalence of ocular demodicosis and ocular surface conditions in patients selected for cataract surgery [4].

Currently, the refractive outcomes of cataract surgery have enabled the use of the procedure for refractive lens exchange. The standard deviation of the new-generation intraocular lens (IOL) calculation formulas in patients undergoing cataract surgery is less than 0.4 D [5], which translates into more than 80% of eyes within 0.5 D target refraction [6]. A single study has even reported ≥88% of eyes within 0.5 D target refraction [7]. An excellent refractive outcome is particularly important due to the constantly growing popularity for spectacle-independence with non-toric and toric premium IOLs. Multifocal intraocular lenses (MIOLs) are used more commonly, to a considerable extent for refractive purposes in non-cataractous eyes [8]. Clear lens exchange and MIOL implantation significantly improve health-related quality of life and vision-related quality of life compared to spectacles in a 1-year follow-up [9,10]. These advancements yielding precise postoperative refractive results have raised patients’ expectations for excellent uncorrected distance visual acuity. 

Complications are still a substantial burden because of the sheer number of cataract surgeries performed worldwide each year. Pseudophakic cystoid macular edema (PCME; Irvine–Gass syndrome) is one of the most common causes of visual impairment after cataract surgery. PCME may occur even in the absence of complications and risk factors. Intraoperative complications, including capsule rupture, vitreous loss, and iris trauma are associated with a higher risk for PCME [11]. Other risk factors include uveitis, poor glycemic control of diabetes [12], and various posterior segment diseases. Currently, there is no common definition for PCME, and therefore, the incidences vary greatly among previously published studies [11]. In the study by Aaronson et al., which was published in this issue, the total incidence of PCME among 536 eyes was 3.5% [13]. Several pre- and postsurgical medical interventions are employed in order to minimize postoperative inflammation and the risk of developing PCME, and these have been a topic of discussion for several years [14]. The results of our previous study have shown findings favoring postoperative topical diclofenac instead of dexamethasone as the first-line postoperative medication after cataract surgery in preventing PCME and suggested reserving dexamethasone as a combination therapy for patients with the highest risk of postoperative complications [14]. Other points of interest when comparing different topical drugs have been the speed of visual recovery and drug tolerability [15]. Importantly, in most cases, PCME is self-limiting using topical nepafenac without any further need for intravitreal treatment. In the study by Aaronson et al., only 1 of the 19 eyes showed no macular edema resolution within two months after topical nepafenac administration [13].

Outcomes of phacoemulsification cataract surgery depend largely on surgeon skills, as well as adherence to a complicated multidrug regimen of anti-inflammatory and antimicrobial therapy [16]. The successful administration of this regimen can be restricted by noncompliance, difficulties in administering eye drops, bioavailability, or side effects. Eliminating the need for eye drop medications in the perioperative period could potentially improve both performance and safety, while also improving patient satisfaction with surgery. Currently, two sustained-release formulations of dexamethasone are available on some markets; the potential options include placement of an anterior chamber suspension in the anterior chamber (Dexycu; Eye-Point Pharmaceuticals, Watertown, MA, USA) or an extended-release intracanalicular insert (Dextenza, OcularTherapeutix, Bedford, MA, USA) [17]. As the introduction of intracameral antibiotics has brought benefits to cataract surgery, intraocular dexamethasone suspension for anterior chamber steroid placement might also assist in improving surgical outcomes. 

Another significant late complication of cataract surgery is posterior capsular opacification (PCO), which is associated with the proliferation and migration of lens epithelial cells [18]. The risk factors for PCO are well known and can be classified as related to the systemic or eye diseases of the patient, to the surgical procedure or to the IOL design [19]. Interventions which have been employed for preventing PCO include modifications in IOL design, the implantation of additional devices and pharmacologic therapy [20]. Sharp posterior optic edges are preferred to round-edged IOLs of the same material for the reduction in PCO and Nd:YAG capsulotomy rates [21]. It is well recognized that although hydrophilic acrylic material is more biocompatible, IOLs made of this material have been shown to support lens epithelial cell adhesion, migration and proliferation and thus PCO development is compared with IOLs made of PMMA or hydrophobic acrylic materials [22,23,24]. A recent economic analysis has highlighted that the choice of IOL for cataract surgery, as a direct consequence of lower Nd:YAG laser capsulotomy rates, may translate into significant savings both financially and with respect to resource allocation for governmental hospitals and the tax-financed national healthcare system [18]. The study by Lindholm et al. has shown that low-diopter IOLs, which are used in myopic patients, are associated with significantly higher risk of Nd:YAG capsulotomy within five years following implantation [25]. Postoperative treatment with steroids among patients undergoing uncomplicated cataract surgery is also associated with lower rates of clinically significant PCO rated compared to treatment with non-steroidal anti-inflammatory drugs [20]. Further studies in this field are warranted and shall potentially further improve the outcomes of cataract surgery.
